# Chronic Lymphocytic Inflammation with Pontine Perivascular Enhancement Responsive to Steroids (CLIPPERS): A Rare Treatable Neuroradiological Spectrum

**DOI:** 10.7759/cureus.4438

**Published:** 2019-04-11

**Authors:** Sachin Khanduri, Harsh Yadav, Nirmal Pandey, Anvit Krishnam, Saket Nigam

**Affiliations:** 1 Radiology, Era's Lucknow Medical College and Hospital, Lucknow, IND; 2 Neurology, Regency Hospital, Kanpur, IND; 3 Radiology, Regency Hospital, Kanpur, IND

**Keywords:** clippers, pons, steroids, punctate enhancement, chronic lymphocytic inflammation with pontine perivascular enhancement responsive to steroids

## Abstract

Chronic lymphocytic inflammation with pontine perivascular enhancement responsive to steroids (CLIPPERS) is a rare CNS inflammatory disorder involving the pons and other parts of the brainstem. It is characterised by a combination of brainstem symptoms and classical magnetic resonance imaging (MRI) features of bilateral, symmetrical punctate, perivascular enhancement of pontine lesions. Another hallmark feature of this rare disease is the responsiveness to corticosteroid treatment. As the corticosteroid treatment is tapered, the symptoms exacerbate and worsen the clinical outcome. Clinicians and radiologists should be aware of this infrequent inflammatory disorder and should always be considered as a differential diagnosis. Herein, we report the case of a 17-year-old female with a similar clinicoradiological spectrum as CLIPPERS.

## Introduction

Chronic lymphocytic inflammation with pontine perivascular enhancement responsive to steroids (CLIPPERS) is a central nervous system (CNS) inflammatory disease characterized by pontine-predominant, punctate, gadolinium-enhancing magnetic resonance imaging (MRI) lesions with exquisite response to corticosteroid therapy first described by Pittock et al. [1]. CLIPPERS has classical clinical, radiological, and pathological findings. Due to the lack of definitive formalised diagnostic markers, there remains a diagnostic uncertainty. This condition features brainstem and cerebellar-related symptoms combined with a characteristic pattern of gadolinium enhancement on MRI. Herein, we report a rare case of CLIPPERS based on clinical and radiological findings.

## Case presentation

A 17-year-old girl presented with a 15-day history of headache (holocranial and predominantly bifrontal) with occasional vomiting and ataxia of gait with no diplopia. She complained of short-lasting episodes of fever for a few days before consulting the doctor. On examination, she had no cranial nerve involvement, no meningeal signs, and a normal fundus examination. She had mild misbalancing on tandem gait. The patient was investigated further and a routine workup was done. Routine hemogram, liver function tests, renal function tests, and serum electrolytes were normal. Serum antinuclear antibody (ANA) and cytoplasmic antineutrophil cytoplasmic antibodies (c-ANCA) levels were normal. Venereal disease research laboratory test (VDRL) and rapid plasma reagin (RPR) antigens were negative. Cerebrospinal fluid (CSF) examination revealed 30 cells (all lymphocytes), an increased protein level of 81 mg/dL (normal range: 12 - 60 mg/dL), and a normal glucose level of 57 mg/dL (normal range: 40 - 70 mg/dL). The CSF examination for fungus and gram stain was negative. No oligoclonal bands were seen. Scrub typhus, Leptospira, dengue, Japanese encephalitis, and toxoplasmosis serologies were negative. Chest computed tomography (CT) and chest x-ray were normal.

**Figure 1:**
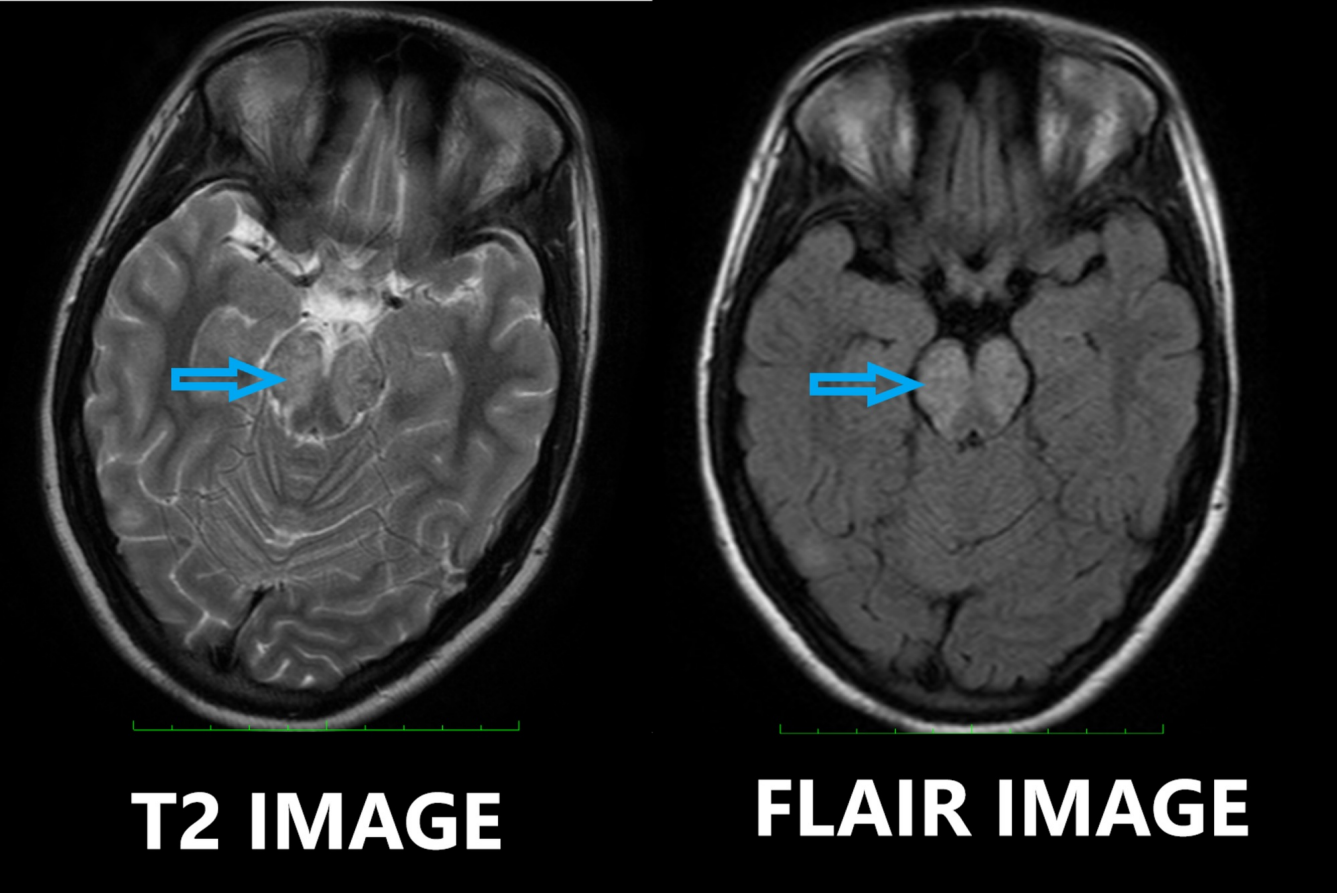
T2-weighted and fluid-attenuated inversion recovery (T2-FLAIR) sequence axial MRI images showing punctate lesions in the pons (blue arrow). MRI: magnetic resonance imaging

**Figure 2:**
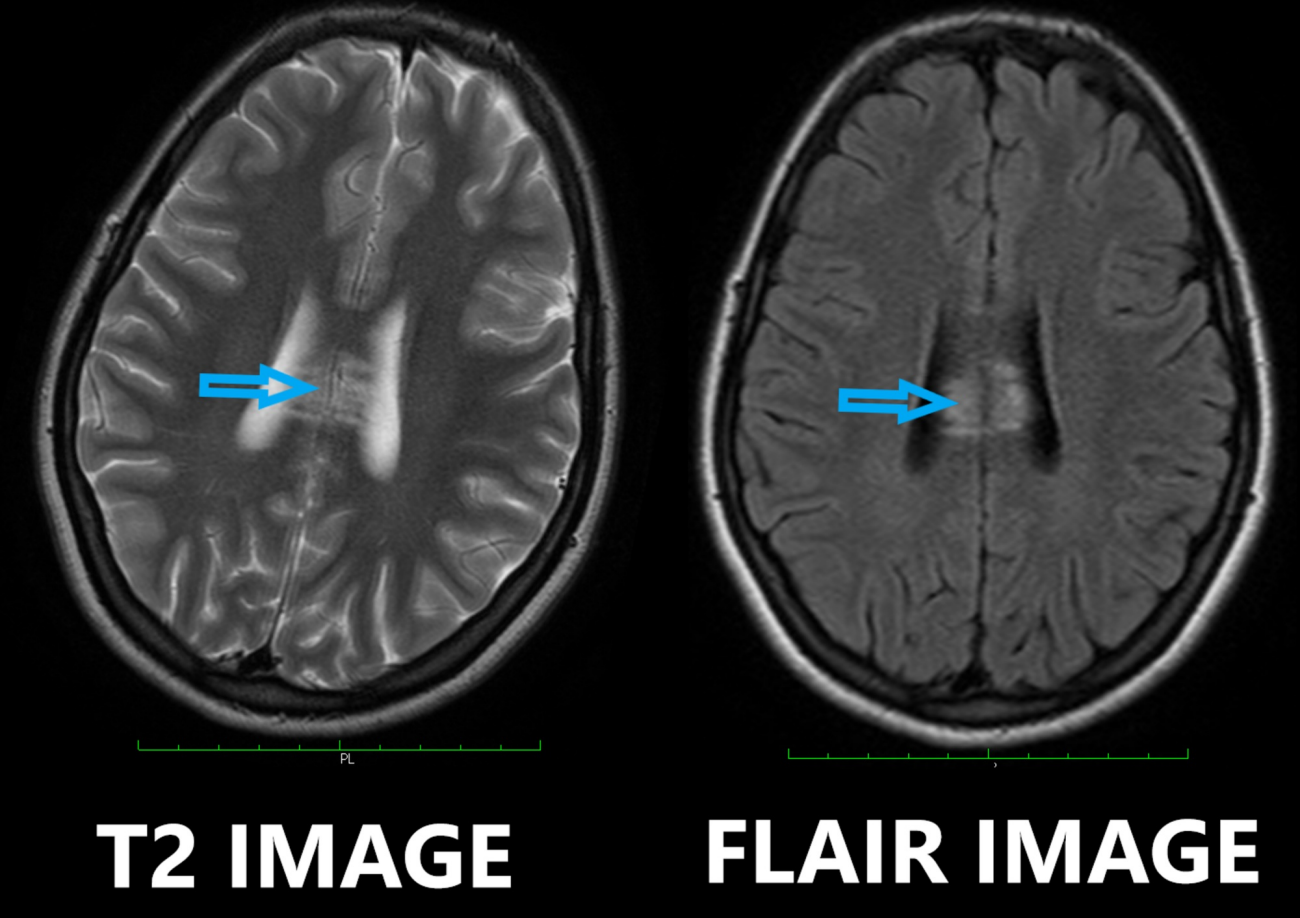
T2-weighted and fluid-attenuated inversion recovery (T2-FLAIR) axial MRI images showing multiple punctate lesions in the corpus callosum (blue arrows) MRI: magnetic resonance imaging

Later, she underwent a contrast-enhanced MRI of the brain which revealed hyperintense T2-weighted/fluid-attenuated inversion recovery sequence (T2-FLAIR) signals involving the midbrain, pons, right cerebellar peduncle, bilateral subthalamic, body and splenium of the corpus callosum, left capsular, and right occipital regions. No restriction on diffusion-weighted imaging (DWI) was seen (Figures 1-2).

**Figure 3:**
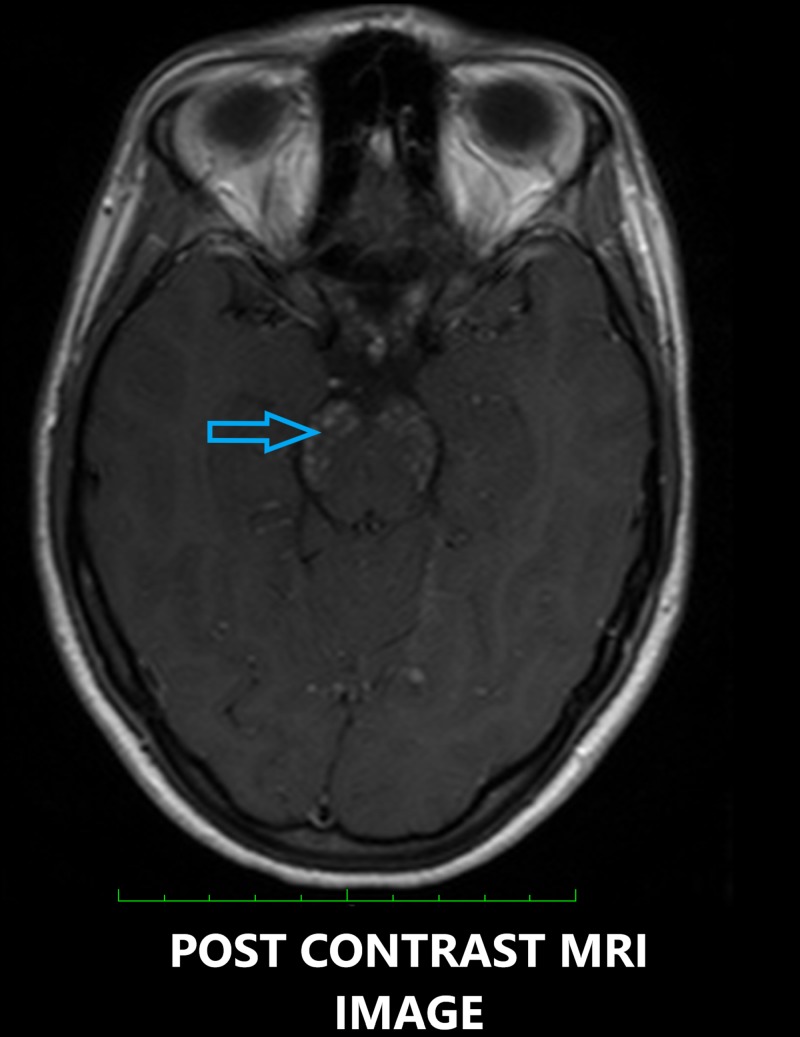
Contrast-enhanced axial magnetic resonance imaging (MRI) showing peppered post-contrast enhancement in the pons (blue arrow).

On contrast enhancement, multiple foci of peppered enhancement were seen in these areas, especially the midbrain, the pons and body, and the splenium of the corpus callosum, distributed in a perivascular pattern (Figure 3).

On the basis of the history, a presumptive diagnosis of acute disseminated encephalomyelitis (ADEM) was made and treatment was started in the form of intravenous (IV) methylprednisolone (1 gram daily for five days). The symptoms of the patient improved, and she was discharged on steroids with no follow-up.

**Figure 4:**
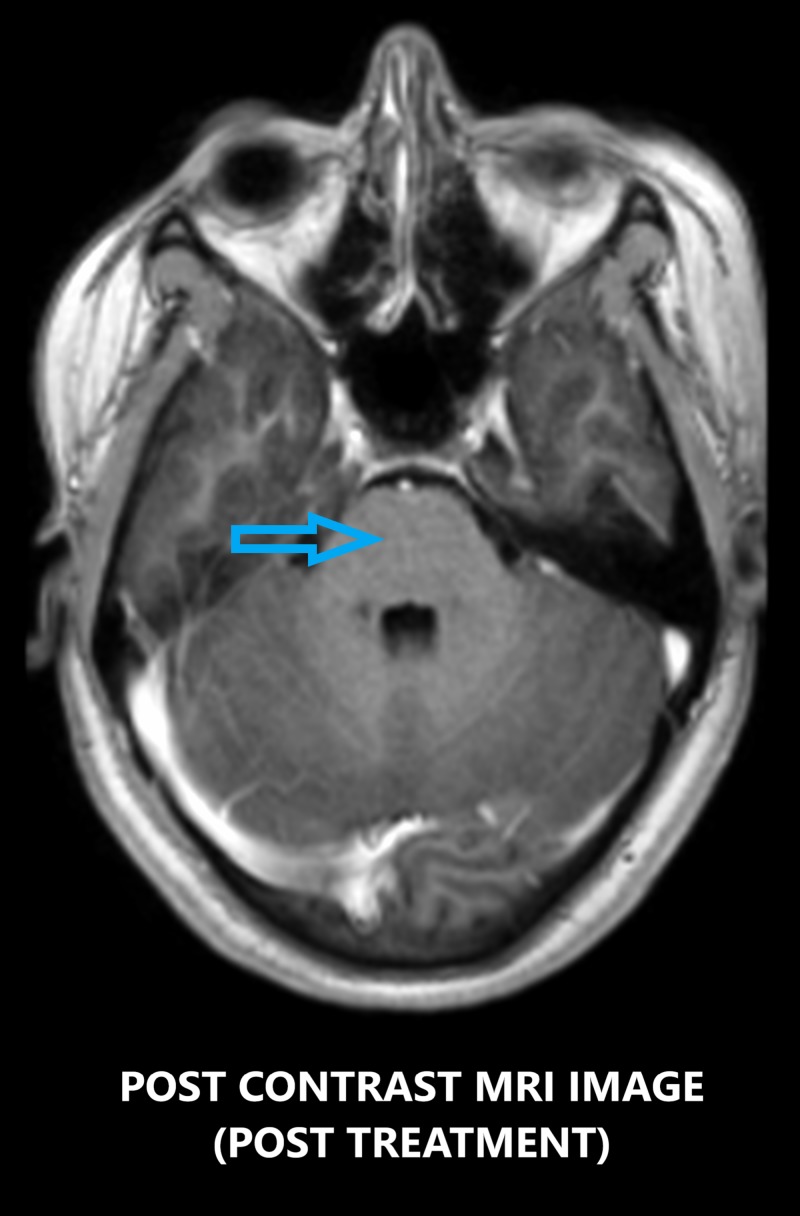
Contrast-enhanced axial MRI image showing the region of the pons after treatment with high-dose steroids depicting no punctate lesions in the brainstem (blue arrow). MRI: magnetic resonance imaging

She was again readmitted after a few days for recurrence of headache and complained of fever for three days prior to admission. She had also complained of right-sided ptosis during the episodes of fever. She was again treated with IV methylprednisolone, 1 gm daily for three days, and she improved completely. A repeat routine hemogram, liver function test (LFT), renal panel, and chest x-ray did not reveal any significant finding. Blood culture was negative. Thyroid profile and Vitamin B12 levels were within normal limits. Again, on recurrence of headaches, she was asked to get a repeat MRI scan of the brain (plain and contrast) which revealed similar findings as the previous MRI done almost a month earlier; however, the lesions had increased in size. The patient’s symptoms and radiological imaging findings responded to treatment with high-dose steroids and methotrexate (Figure 4), but after several trials to taper the dose of steroids, the symptoms reappeared and were progressive in nature.

## Discussion

CLIPPERS is a rare chronic inflammatory neurological syndrome affecting multiple regions of the brain, including the brainstem, cerebellum, and spinal cord, that has gained recognition since its introduction in 2010 by Pittock [1]. The hallmark finding of CLIPPERS is the presence of multiple punctate, curvilinear lesions, notably showing a “peppered” appearance of the pons and other parts of the brainstem on a contrast-enhanced MRI [2], with relative sparing of the supratentorial brain. Similar punctate-type enhancement has also been described in the basal ganglia, corpus callosum, and spinal cord [3]. Positive clinical and radiological response to corticosteroids is observed. Other differential diagnoses include neurosarcoidosis, vasculitis, lymphoma, and encephalitis.

CLIPPERS usually manifests between 13 to 78 years of age [1, 4] with no predilection to either sex. The usual presenting symptoms include progressive gait disorders, ataxia, and dysarthria. Other symptoms include facial paresthesia, diplopia, and myelopathy [5].

 Pathologically, it is characterised by marked CD3-positive T-cell predominant lymphocytic perivascular inﬂammation with co-existent white and grey matter, as well as meningeal inﬂammation with the absence of vessel wall involvement [5-6].

 A definitive diagnostic criterion of CLIPPERS requires clinical, radiological, and neuropathological evidence.

Clinically, patients with CLIPPERS show signs of pontocerebellar dysfunction with improvement in treatment by using steroids. Radiologically, CLIPPERS is defined by the presence of multiple punctate lesions showing a ‘peppered’ enhancement on post-gadolinium scans in the pons, cerebellum, corpus callosum, and spinal cord. Typically, the lesions in the pontine region are multiple and bilateral with the size < 3 mm in diameter [1]. Enhancing lesions > 3 mm in size are typically nodular [3]. Bilateral location of disease is an important feature pointing towards CLIPPERS as the diagnosis. Patients with unilateral enhancing lesions should be carefully evaluated for alternate causes, such as neoplasms or granulomatous diseases [6]. On corticosteroid treatment, there is a significant reduction in the enhancement and size of the pontine lesions. On pathology, CLIPPERS shows dense lymphocytic inﬂammation with perivascular predominance and parenchymal diffuse inﬁltration; both white matter and grey matter could be involved. T-cells predominating inﬁltration (CD4 > CD8) with variable macrophage components are usually seen. Lymphocytes are predominantly CD3-positive; however, CD4-positive lymphocytic infiltration has been described [5]. The usual treatment course of CLIPPERS reports maintenance of remission using hydroxychloroquine, azathioprine, and even antitubercular drugs [7-9]. A recommended five-day course of daily intravenous methylprednisone, followed by prolonged treatment of 1 mg/kg of oral prednisone daily [1], with appropriate precautions regarding infection prevention, pneumocystis pneumonia prophylaxis, osteoporosis prevention, and weight management is followed. Steroid-sparing agents, such as methotrexate or azathioprine, can be started at the same time [10] as the oral prednisone and typically require higher doses. The disease is known to have a relapsing/remitting course, for which weekly methotrexate is added to treat the relapse event. Oral steroid treatment leads to complete resolution of the enhancing lesions with gradual atrophy of the affected regions and resolution of clinical symptoms. 

Although CLIPPERS has proposed definitive diagnostic criteria, a lack of definitive biomarkers and non-availability of a brain biopsy in all suspected cases remains a clinical challenge.

In the current study, we report a rare case of CLIPPERS with definitive diagnostic clinical and radiological features showing remission and improvement on steroids.

## Conclusions

CLIPPERS is a CNS inflammatory disease characterised by marked perivascular pontine-enhancing lesions on MRI showing responsiveness to high-dose steroids. CLIPPERS is diagnosed using clinical history, neuroimaging, and biopsy. Although long-term treatment with high-dose steroids can pose numerous side effects, long-term treatment of CLIPPERS is successful with a variety of steroid-sparing drugs, suggesting that long-term outcome is good if early use of immunosuppressive drugs is started. In our case, the diagnosis of CLIPPERS was mainly based on clinical history and neuroimaging, both in the pre- and post-treatment phases.
